# Impact of COVID-19 pandemic policies on ADHD medication prescriptions among children and adolescents in Portugal

**DOI:** 10.1007/s00431-025-06229-y

**Published:** 2025-06-12

**Authors:** Célia Silva, Mariia Melnikova, Rui Santos Ivo, Cláudia Furtado

**Affiliations:** 1 Information and Strategic Planning Department (DIPE), INFARMED, National Authority of Medicines and Health Products, I.P., Lisbon, Portugal; 2 INFARMED, National Authority of Medicines and Health Products, I.P, Lisbon, Portugal; 3 NOVA National School of Public Health, NOVA University Lisbon, Lisbon, Portugal; 4 Lisbon, Portugal

**Keywords:** ADHD, COVID-19 pandemic, Medicine prescription, Paediatric, Interrupted time series

## Abstract

This study aims to analyse the prescription of medication for attention-deficit/hyperactivity disorder (ADHD) in children and adolescents, by age group and gender, before and during the COVID-19 pandemic, as well as the impact of government policies during this period. This study was a retrospective cohort analysis using data from the Portuguese national prescription database and included all children and adolescents aged 5–19 years. We conducted an interrupted time series analysis, modelled using a seasonal autoregressive integrated moving average (SARIMA) approach, to evaluate the impact on prescription medications while considering two intervention points resulting from government policies: March 2020, marking the start of strict anti-pandemic measures, and May 2021, marking the beginning of their easing. Following the start of strict anti-pandemic measures, from March 2020 to May 2021, a 9% lower prescription rate than expected was observed, while from June 2021 to December 2023, the prescription rate was greater than expected (+ 32%, with a peak of + 41% in children 5–9 years old) and more pronounced in girls. *Conclusions*: These findings highlight the complex impact of the pandemic on different age groups and between genders on the prescription of ADHD medicines.

**Table:** 

**What is Known:** • *ADHD medications are considered effective and have been increasingly prescribed to children and adolescents in recent years.*
**What is New:** • *Following the COVID-19 pandemic, the increase in ADHD medication prescriptions exceeded expected trends.* • *Stratification by age group and gender revealed gender differences, with a more pronounced rise in prescriptions observed among girls.*

**Supplementary Information:**

The online version contains supplementary material available at 10.1007/s00431-025-06229-y.

## Introduction

Attention-deficit/hyperactivity disorder (ADHD) is a neurodevelopmental disorder defined by persistent, impairing, and developmentally inappropriate inattentive/disorganised and/or hyperactive/impulsive behaviours that lie at the far end of a normally distributed continuum [1]. It affects individuals of all ages, with an estimated global prevalence that has been steadily increasing in recent years [2], with gender differences expressed by higher prevalence rates in boys than in girls [3]. ADHD poses significant challenges in various aspects of an individual’s life, including academic performance, social interactions, and overall well-being [4].

The diagnosis and treatment of ADHD involve a multidimensional approach, considering both pharmacological and non-pharmacological interventions [5]. While there are established diagnostic criteria, variations exist in the approaches used by clinicians and researchers worldwide [1]. Pharmacological interventions, such as stimulant medications (e.g. methylphenidate, lisdexamphetamine), non-stimulant medications (e.g. atomoxetine), and other adjunctive therapies, are commonly prescribed to manage ADHD symptoms [6].

Evidence supports medication treatments for managing ADHD symptoms and improving functional outcomes across different age groups. Some studies have shown that certain medications, such as methylphenidate, are preferred as a first-choice medication for children and adolescents, while amphetamines are preferred for adults [7]. Another study indicates that methylphenidate significantly improves ADHD symptoms and behaviour in children and adolescents compared to placebo or no-intervention [8]. Guidelines emphasise the importance of medication interventions alongside non-pharmacological treatments [9, 10], and research on routine care non-pharmacological treatment for adolescents with ADHD supports a multifaceted approach [11]. The effectiveness of specific treatments may vary depending on the age of the children and the characteristics of the treatment [12]. Furthermore, medication treatments have been associated with decreased risks of ADHD-related functional outcomes, highlighting the significance of early diagnosis and treatment efforts [13]. These findings collectively affirm the crucial role of medication treatments in managing ADHD symptoms and enhancing functional outcomes.

However, the COVID-19 pandemic has brought unprecedented challenges to the healthcare system and has had a significant impact on individuals’ mental health worldwide. The pandemic and the associated measures, such as lockdowns and social distancing, have affected children and adolescents with ADHD in various ways [14]. In addition, in several European countries, changes in routine clinical practice were implemented to minimise the transmission of COVID-19 [15], which may have impacted access to mental health care for children and adolescents.

In relation to mental health, some reports suggest that the pandemic-induced changes have led to improved symptomatology and well-being among children with ADHD, possibly due to reduced academic pressures and more flexible routines [16]. Conversely, other studies have reported a range of difficulties and challenges faced by children with ADHD during the pandemic, including increased anxiety, depression, irritability, mood swings, inattention, and sleep disturbances [17, 18].

A recent study examining ADHD medication consumption in overall population during the pandemic found that, on average, consumption increased by 1.60% per country, and the deviations in consumption strongly correlated with the stringency of anti-pandemic government policies [19]. The same author study examined ADHD medication consumption in Europe from 2020 to 2022, compared to the predicted consumption across 28 European countries. It concluded that it exceeded predictions by an average of 16.4% at the country level [20]. However, these studies compared sales volumes of ADHD medication and its evolution using DDD overall population.

These findings underscore the complex interplay between the COVID-19 pandemic, government policies, access to health care, and ADHD medication consumption.

Despite growing interest, research on the long-term effects of COVID-19 lockdowns, home-schooling, and social distancing on children and adolescents with ADHD remains limited. This study investigates the impact of the COVID-19 pandemic on ADHD medication prescriptions, stratified by age group and gender. To our knowledge, this is the first study to address possible differences in the impact of the COVID-19 pandemic on ADHD prescribing across gender and age groups.

By analysing prescription data from all children and adolescents in Portugal, we aim to provide insights into the changes, if any, in the utilisation of pharmacological interventions for ADHD during and after the pandemic, and differences across gender and age.

## Methods

Portugal has universal health coverage through its publicly funded National Health Service. The public sector is complemented by private practice and the social sector. Prescriptions from both public and private practices are reimbursed through public funding.

The analysis was based on the medication prescription data from the Portuguese National Database of Medical Prescriptions, which covers approximately 96% of all prescriptions nationwide. Medicine prescriptions were categorised according to the World Health Organization’s Anatomical Therapeutic Chemical (ATC) classification system, with the analysis conducted at the level of chemical substances (ATC 5 th level).

This study includes all prescriptions corresponding to three ATC 5 th level codes commonly used in the treatment of ADHD: methylphenidate (N06BA04), lisdexamfetamine (N06BA12), and atomoxetine (N06BA09). Prescriptions for methylphenidate, lisdexamfetamine, and atomoxetine were selected and extracted for each month of the pre-pandemic and COVID-19 impact period (1 January 2016 to 31 December 2023) and do not contain individual-level data. We extracted the number of prescribed packages grouped by the above three substances, gender, and age groups. The study focused on three age subgroups: 5–9, 10–14, and 15–19 years. Prescription settings were aggregated by public sector (primary care and hospital settings), private sector (private medical practice, clinics and private hospitals), and the social sector (clinics and social hospitals). Prescriptions outside the desired age range were excluded from the analysis.

### Outcomes

The main outcome of interest was the number of prescribed packages per 1000 inhabitants per month. To determine the number of prescribed packages per child and adolescent, we used the resident population per year, as reported by Statistics Portugal [21], as the denominator. We calculated the rate of prescribed packages per 1000 children and adolescents, stratified by age group and gender.

### Study design

An interrupted time series was used to compare prescription rates of ADHD medicines before and during the pandemic considering two intervention points resulting from government policies: March 2020 [22], marking the start of strict anti-pandemic measures, and May 2021 [23], marking the beginning of the easing of these measures. March 2020 and May 2021 were selected as change points, as they marked the approval of significant legislation and government policies that could potentially influence medication prescribing patterns.

### Statistical analysis

We performed an interrupted time series analysis modelled by a seasonal autoregressive integrated moving average (SARIMA), with exogenous variables [24–28], with monthly data to assess the effect on prescribing ADHD medicines. We had 96 consecutive monthly data points available, with the first interruption point set at the 51 st month (March 2020) and the second interruption point at the 65 th month (May 2021). Autocorrelation was adjusted using SARIMA models. We used an automated algorithm, specifically auto.arima in the forecast package for R [29, 30], to identify the SARIMA model terms. The default fitting method provided by auto.arima first employs conditional-sum-of-squares to obtain starting values and then applies maximum likelihood estimation for the final parameter estimates. We pre-specified a maximum of one order of differencing operations to create stationary series for modelling; the selection of the other model parameters was all determined by minimising the AICc (Corrected Akaike’s Information Criterion). To assess changes in prescriptions during the COVID-19 outbreak and the consequent preventive and protective measures, we hypothesised an immediate drop in prescriptions in March 2020 (step change), along with a change in slope. We tested pre-pandemic trends and examined the change in trend brought about by the preventive and protective policies implemented by the Portuguese government in response to the COVID-19 pandemic [28]. Missing data accounted for 1.5% of all prescriptions during the study period and were related to gender. These cases were handled using listwise deletion. To identify potential outliers in the time series, we applied the *Z*-score method, considering observations with absolute *Z*-scores greater than 2.5 as extreme values. An extreme fluctuation was observed in November 2023, present in all-time series except for the age groups 10 to 14 and 15 to 19. No adjustments were made to this extreme value. The analysis was carried out in R Studio version 2023.06.0 [31] based on R version 4.3.0 [32] and packages [33–38]. The interrupted time series analysis is a quasi-experimental design that does not necessitate covariate adjustment, and therefore, no adjustments for confounders were made.

### Ethical considerations

This study adhered to the General Data Protection Regulation (EU 2016/679), ensuring that only aggregated, non-identifiable data was accessed and used, thus not requiring formal ethical approval.

## Results

From January 2016 to December 2023, approximately 138,000 children and adolescents aged 5–19 years in Portugal had at least one ADHD prescription, with 31% being female and 69% male; this represents around 9% of the resident population in this age group. The age group with the highest number of prescribed packages was 10–14 years (47.3%), followed by the age groups 5–9 (26.4%) and 15–19 (26.3%). The most frequently prescribed medicine for ADHD in 2023 was methylphenidate (79%), followed by lisdexamfetamine (18%), and atomoxetine (3%).

We observed changes in the percentage of prescriptions by setting after March 2020: the private sector had an increase of 4 percentage points, while the public sector experienced a decrease of the same magnitude. Additionally, the third sector remained relatively stable, accounting for only 1% of prescriptions throughout the study period. In 2023, the number of prescribed packages by physicians in the public sector accounted for 67%, the private sector 32%, and the third sector 1%.

As expected, a seasonal pattern of the prescription is evident, with declines during the summer school holidays (June, July, and August) and increases afterwards, particularly at the start of the school year and peaking at the beginning of the second school term (January). Throughout the analysed period, 2023 showed the highest monthly rates, peaking in November. It is also evident that the monthly prescription rate is higher from April 2021 to December 2023 (see Supplementary Information Fig. 1 for seasonal plots with age group details).

### Prescription before and during COVID-19 pandemic

The effect of the COVID-19 pandemic on overall ADHD prescriptions is shown in Table 1. Up until February 2020, across all age groups, there was a monthly mean of 16.7 packages per 1000 children. Between March 2020 and April 2021, the monthly mean rose to 17.9 per 1000, an approximate 8% increase. After April 2021, we observed a 59% increase in the monthly mean (26.9 per 1000). The age group 5–9 years had the greatest increase, at approximately 82%.

**Table 1:** Descriptive statistics for monthly rates, per age group, standard deviation (STD), 95% confidence intervals (CI), and the percentage change during preventive and protective policies (change point 1) and after the withdraw of the restrictive policies (change point 2)

Packages per month ^x^	Mean	STD	95% CI	% Change
All age groups				
Before COVID-19 pandemic ^a^	16.7	7.6	(14.5; 18.8)	-
After the first change point ^b^	17.9	5.7	(14.6; 21.2)	7.6
After the second change point ^c^	26.9	8.2	(24.0; 29.8)	58.9
Children aged 5–9				
Before COVID-19 pandemic ^a^	12.1	4.9	(10.7; 13.5)	-
After the first change point ^b^	13.1	4.2	(10.7; 15.6)	8.6
After the second change point ^c^	22.3	8.4	(19.3; 25.4)	81.6
Adolescents aged 10–14				
Before COVID-19 pandemic ^a^	27.1	12.8	(23.5; 30.8)	-
After the first change point ^b^	27.6	9.3	(22.3; 33.0)	1.9
After the second change point ^c^	39.4	11.7	(35.2; 43.6)	44.5
Adolescents aged 15–19				
Before COVID-19 pandemic ^a^	10.8	5.7	(9.2; 12.4)	-
After the first change point ^b^	12.8	4.1	(10.4; 15.2)	18.3
After the second change point ^c^	19.1	6.1	(16.9; 21.3)	69.9

^x^Per 1000 children/adolescents. ^a^From January 2016 to February 2020. ^b^From March 2020 to April 2021. ^c^From May 2021 to December 2023 (percentage change compared with the period from January 2016 to April 2021)

Before COVID-19 pandemic, the results suggested a monthly increase of 0.3 per 1000 children (95% CI 0.2; 0.4). The first policy change in March 2020 triggered a step decrease of 7.9 per 1000 (95% CI − 12.5; − 3.5), followed by a monthly increase of 0.4 per 1000 (95% CI 0.3; 0.5). After the first policy change, all age groups experienced an immediate step decrease in prescriptions and a change in trend, with the greatest change observed in the 5–9 age group (Table 2).

**Table 2:** Regression with SARIMA models. Coefficients: estimated change in prescription volumes per age group before and after the first policy change

Packages per month ^x^	Value	Standard error	95% CI
All age groups			
Trend before COVID-19 pandemic ^a^	0.3	0.1	(0.2; 0.4)
First change point ^b^	− 7.9	2.3	(− 12.5; − 3.4)
Trend after the first change point ^c^	0.4	0.1	(0.3; 0.5)
Children aged 5–9			
Trend before COVID-19 pandemic ^a^	0.2	0.1	(0.1; 0.3)
First change point ^b^	− 6.3	1.7	(− 9.6; − 3.0)
Trend after the first change point ^c^	0.4	0.1	(0.3; 0.5)
Adolescents aged 10–14			
Trend before COVID-19 pandemic ^a^	0.5	0.1	(0.2; 0.7)
First change point ^b^	− 12.0	3.9	(− 19.7; − 4.3)
Trend after the first change point ^c^	0.5	0.1	(0.2; 0.7)
Adolescents aged 15–19			
Trend before COVID-19 pandemic ^a^	0.2	0.0	(0.2; 0.3)
First change point ^b^	− 5.6	1.6	(− 8.7; − 2.5)
Trend after the first change point ^c^	0.3	0.1	(0.2; 0.4)

^x^Per 1000 children/adolescents. ^a^From January 2016 to February 2020. ^b^March 2020. ^c^From April 2020 to December 2023

### Gender differences

As expected, boys were more likely to be prescribed ADHD medicines than girls, but after COVID-19 outbreak, the increase in prescribing was higher for girls than for boys. For girls, we observed a 72% increase in the monthly mean of prescriptions across all age groups; for boys, the increase was 55% (Table 3). The percentage change for boys and girls aged 5–9 was similar (86% for girls and 80% for boys). The greatest increase in percentage change was seen among girls aged 15–19, with 102%, while the greatest increase in volume was observed among boys aged 10–14 (Supplementary Information Table 1).

**Table 3:** Descriptive statistics for monthly rates, per gender, standard deviation (STD), 95% confidence intervals (CI), and the percentage change during preventive and protective policies (change point 1) and after the withdraw of the restrictive policies (change point 2)

Packages per month ^x^	Mean	STD	95% CI	% Change
Girls (all age groups)				
Before COVID-19 pandemic ^a^	8.7	4.0	(7.5; 9.8)	-
First change point ^b^	9.4	3.2	(7.5; 11.3)	8.6
Second change point ^c^	15.2	5.0	(13.4; 17.0)	72.1
Boys (all age groups)				
Before COVID-19 pandemic ^a^	24.3	11.1	(21.1; 27.4)	-
First change point ^b^	26.1	8.2	(21.4; 30.8)	7.5
Second change point ^c^	38.1	11.3	(34.1; 42.2)	54.5

^x^Per 1000 children/adolescents. ^a^From January 2016 to February 2020. ^b^From March 2020 to April 2021. ^c^From May 2021 to December 2023 (percentage change compared with the period from January 2016 to April 2021)

Table 4 suggests steadily growing monthly parameter estimates for prescriptions before the pandemic for both genders, and are followed by a negative step change after the first policy change in March 2020. Following March 2020, there was a monthly increase, higher than before the pandemic, for both genders.

**Table 4:** Regression with ARIMA models. Coefficients: estimated change in prescription volumes per gender before and after the first policy change

Packages per month ^x^	Value	Standard error	95% CI
Girls (all age groups)			
Trend before COVID-19 Pandemic ^a^	0.2	0.0	(0.1; 0.2)
First change point ^b^	− 5.4	1.2	(− 7.7; − 3.1)
Trend after the first change point ^c^	0.3	0.0	(0.2; 0.4)
Boys (all age groups)			
Trend before COVID-19 pandemic ^a^	0.4	0.1	(0.3; 0.6)
First change point ^b^	− 11.2	3.4	(− 17.8; − 4.6)
Trend after the first change point ^c^	0.5	0.1	(0.3; 0.7)

^x^Per 1000 children/adolescents. ^a^From January 2016 to February 2020. ^b^March 2020. ^c^From April 2020 to December 2023

### What if there was no COVID-19 pandemic? predicted vs. observed trends

**Fig. 1:**
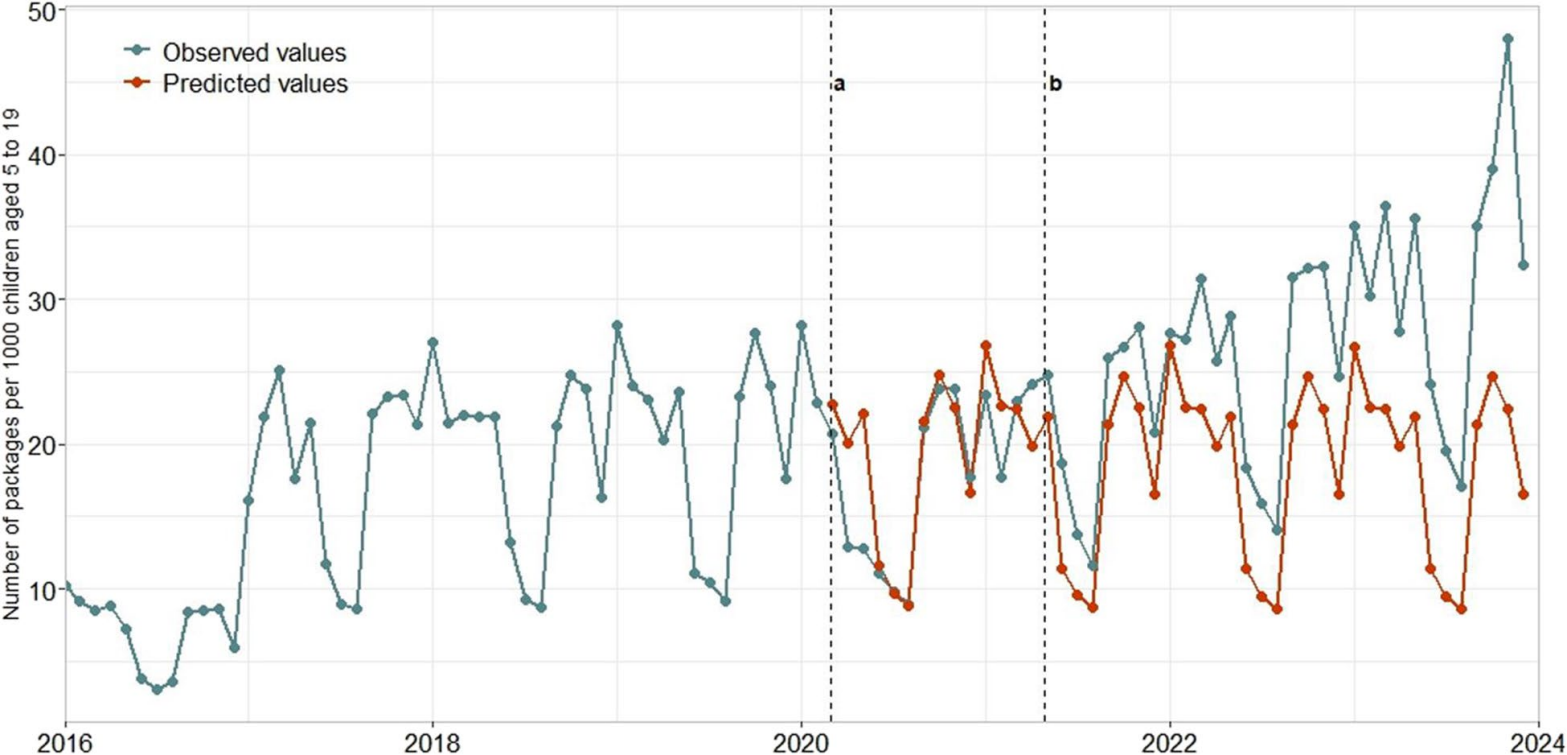
Observed and predicted values per all age groups: predicted values after March 2020 and after May 2021. **a** = March 2020: COVID-19 pandemic protection policies. **b** = May 2021: start of relief of restrictive policies

To understand the impact of the two policy changes due to COVID-19 pandemic, we calculated the difference between the observed results for ADHD prescription and the predicted results from our models (Fig. 1) which consider the scenario “what if there was no COVID-19 pandemic?”.

After the first policy change, and over a 14-month period across all age groups, we observed a lower prescription rate than we predicted (on average, 9% less), while after the second policy change and over 32 months, we observed a higher prescription rate than we predicted (on average, 32% more).

The age group most affected during the first policy change was 10–14, with a prescription rate 10% lower than predicted. During the second policy change, the age group 5–9 had the greatest increase in the prescription rate, with observed rates 41% higher than predicted.

By gender, we observed that after the first policy change and during a 14-month period, there was a lower prescription rate than we predicted (on average, 12% less for girls and 9% less for boys). However, after the second policy change, we observed a higher prescription rate than predicted (on average, 33% more for girls and 29% more for boys).

**Fig. 2:**
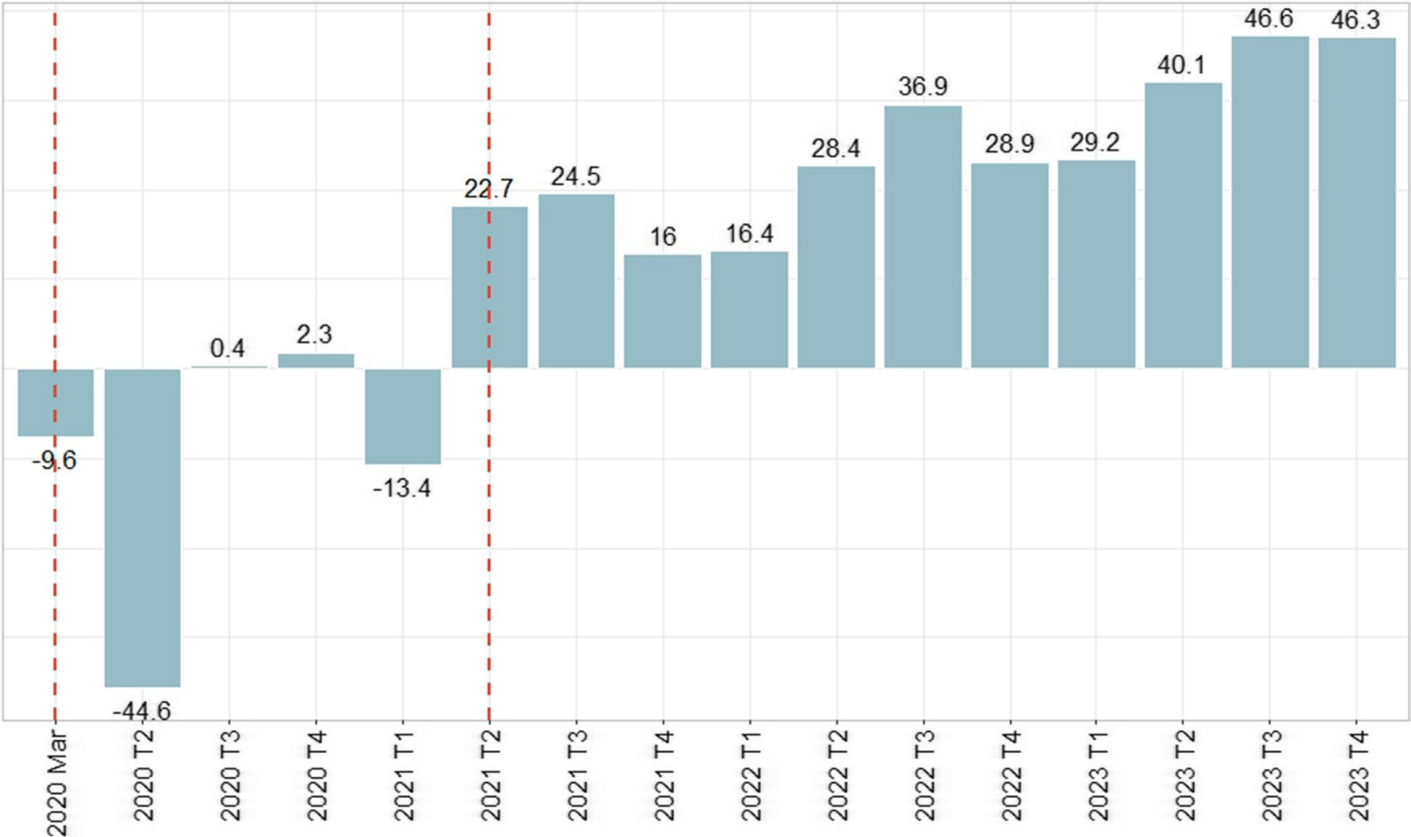
Average relative difference between observed and predicted ADHD medicine’s prescription rate, per trimester. Dashed orange line: First and second policy change

Figure 2 shows the relative average differences between the observed and predicted rates of ADHD prescriptions, aggregated by quarter. The prescription rate began to drop immediately after the first policy change, continuing into the second quarter of 2020, where the decline was greatest, at approximately 45%. In the following two quarters, the average loss diminished, and in the first quarter of 2021, it dropped again, by approximately 13% (during the second national lockdown).

After the second policy change, in the second quarter of 2021, observed prescriptions began to exceed the predicted rate, and in the last quarter of the analysis period, the observed prescription rate was on average 46% greater than predicted.

## Discussion

Despite its importance, not much is known about the long-term mental health effects of the COVID-19 pandemic on children and adolescents, which underscores the importance of monitoring access to mental health care, and in this particular case, prescription of ADHD medication.

This nationwide study demonstrated an increase in ADHD medication prescriptions among children and adolescents aged 5–19 years in Portugal between 2016 and 2023. The sharp rise in prescriptions following the reduction of COVID-19 restrictions underscores the complex interplay between public health policies, educational disruptions, and the behavioural needs of children during this period.

Our analysis revealed that strict pandemic measures were associated with an immediate decline in prescriptions, possibly due to limited access to healthcare services and reduced academic demands. Disruptions to care-seeking behaviours and preventive interventions were observed across the majority of European countries, including Portugal [39], where several appointments were cancelled or postponed. In Portugal, as in many other European countries, the initial months of the COVID-19 pandemic were marked by significant restrictions on in-person appointments due to mobility limitations and safety concerns. However, there was a corresponding increase in non-face-to-face appointments [40], which helped mitigate some of the barriers to accessing treatment during this period.

The subsequent surge in prescriptions post-2021, consistent with the findings of Gimbach et al. [19, 20] and also of Khan and Hassan [41], although their analyses were based on dispensing data in the general population, may reflect the exacerbation of mental health burdens during the pandemic among children and adolescents with ADHD symptoms, driven by school closures, social distancing measures, and decreased levels of physical activity [42]. The impact of pandemic measures appears to have affected adolescent mental health beyond ADHD, as evidenced by the study conducted by Pedersen et al. [43]. Their research found that, among adolescents, the pandemic was associated with an increased trend for ADHD medications, antidepressants, and anxiolytics in Norway and Sweden, but not in Italy.

This rise in prescriptions may also reflect heightened parental concerns, school reintegration challenges, and increased recognition of ADHD symptoms by educators and healthcare providers. Given the limited access to publicly funded psychological therapies within the National Health Service, pharmacological interventions may have become increasingly central in the management of ADHD. Additionally, healthcare activity increased, as evidenced by a higher number of medical appointments in both primary and hospital care during 2021, 2022, and 2023, which may have contributed to a rise in diagnoses and prescriptions [44–47].

The findings reveal significant age and gender variations, with boys aged 10–14 showing the highest prescription rates, consistent with established patterns of ADHD diagnosis [3]. However, girls experienced a greater relative increase, potentially due to historical underdiagnosis, and heightened emotional challenges during the pandemic [48–50]. In line with this, the 2022 Health Behaviour in School-aged Children (HBSC) study showed an increase in school pressure from 2018 to 2022 in many countries and regions on older adolescents, particularly girls [51], which may explain some of these results. Additionally, more girls than boys reported a negative COVID-19 impact on their mental health in nearly all 22 countries and regions, based on the same survey [52]. In accordance with a recent Finnish study, the prevalence of ADHD differs by gender and was higher in males, but the relative growth was higher in females compared to males [53]. These results highlight the need for a differentiated clinical approach to ADHD in girls, who may frequently show internalised symptoms [54], with the aim of reducing underdiagnosis.

While medications remain a cornerstone of ADHD treatment, overreliance on pharmacological interventions may lead to over-medication. The observed trends highlight the need for multifaceted and balanced interventions rather than relying solely on pharmacological treatments. Behavioural therapies, such as parent training programs and school-based support, are critical complements to pharmacological treatments [55, 56]. Developing integrated care pathways that include psychoeducation for parents, individualised educational plans, and community-based resources could help mitigate the challenges amplified during the pandemic [56, 57]. These interventions can address the social and emotional needs of children while reducing reliance on medication alone.

This study has some limitations. The reliance on prescription data may not fully capture the nuances of ADHD management, such as the extent of non-pharmacological treatments these children and adolescents were subjected to. However, a major strength of this study is its nationwide coverage of the paediatric population in Portugal, as well as its reliance on routinely collected data, which are less prone to bias than survey-based data.

In conclusion, the study underscores significant changes in ADHD medication during the COVID-19 pandemic. The findings highlight the importance of a comprehensive treatment approach, incorporating psychosocial and educational interventions alongside pharmacological options. These insights can inform policies and practices to better support children with ADHD in a post-pandemic world.

## Supplementary Information

Below is the link to the electronic supplementary material.

Supplementary file1 (PDF 94.3 KB)

## Data Availability

Dataset D1: Number of prescribed packages of ADHD's medicines to children and adolescents in Portugal from January 2016 to December 2023. Raw data are not publicly available to preserve individuals’ privacy under the European General Data Protection Regulation. Data requests can be made to INFARMED via this email cimi@infarmed.pt mentioning this study. Dataset D2: Number of the resident population in Portugal (Mainland and Islands) from 2016 to 2023, by sex and age group, can be obtained at Statistics Portugal (https://www.ine.pt) with the selection of"Resident population (Long series, start 1991)".

## Abbreviations

ADHD: Attention-deficit hyperactivity disorder
ARIMA: Autoregressive integrated moving average
ATC: Anatomical Therapeutic Chemical
CI: Confidence interval
STD: Standard deviation
