# Supplementary material for: Impact of COVID-19 pandemic policies on ADHD medication prescriptions among children and adolescents in Portugal
Source: Eur J Pediatr. 2025 Jun 12;184(7):415. doi: 10.1007/s00431-025-06229-y (PMC12158850; doi:10.1007/s00431-025-06229-y)
Supplement: Supplementary file 1 — Supplementary file1 (PDF 94.3 KB) [file 431_2025_6229_MOESM1_ESM.pdf]

Title

Impact of COVID-19 pandemic policies on ADHD medication prescriptions among children and adolescents in Portugal

Journal name

European Child & Adolescent Psychiatry

Author information

Name: Célia Silva

Affiliation: INFARMED, National Authority of Medicines and Health Products, I.P., Information and Strategic Planning Department (DIPE), Lisbon, Portugal

E-mail: celia.silva@infarmed.pt

ORCID: 0009-0007-8819-3749

Name: Mariia Melnikova, MD

E-mail: mariia.melnikova.md@gmail.com

ORCID: 0009-0009-3381-2025

Name: Rui Santos Ivo

Affiliation: INFARMED, National Authority of Medicines and Health Products, I.P., Lisbon, Portugal

E-mail: rui.ivo@infarmed.pt

ORCID: 0000-0001-8400-0013

Name: Cláudia Furtado,

Affiliation: INFARMED, National Authority of Medicines and Health Products, I.P., Information and Strategic Planning Department (DIPE), Lisbon, Portugal. NOVA National School of Public Health, NOVA University Lisbon, Lisbon, Portugal

E-mail: claudia.furtado@infarmed.pt

ORCID: 0000-0002-6754-9319

Supplementary Information

Supplementary Information Fig 1. Seasonal plots for monthly prescribed packages per 1000 children overall age groups and by age group

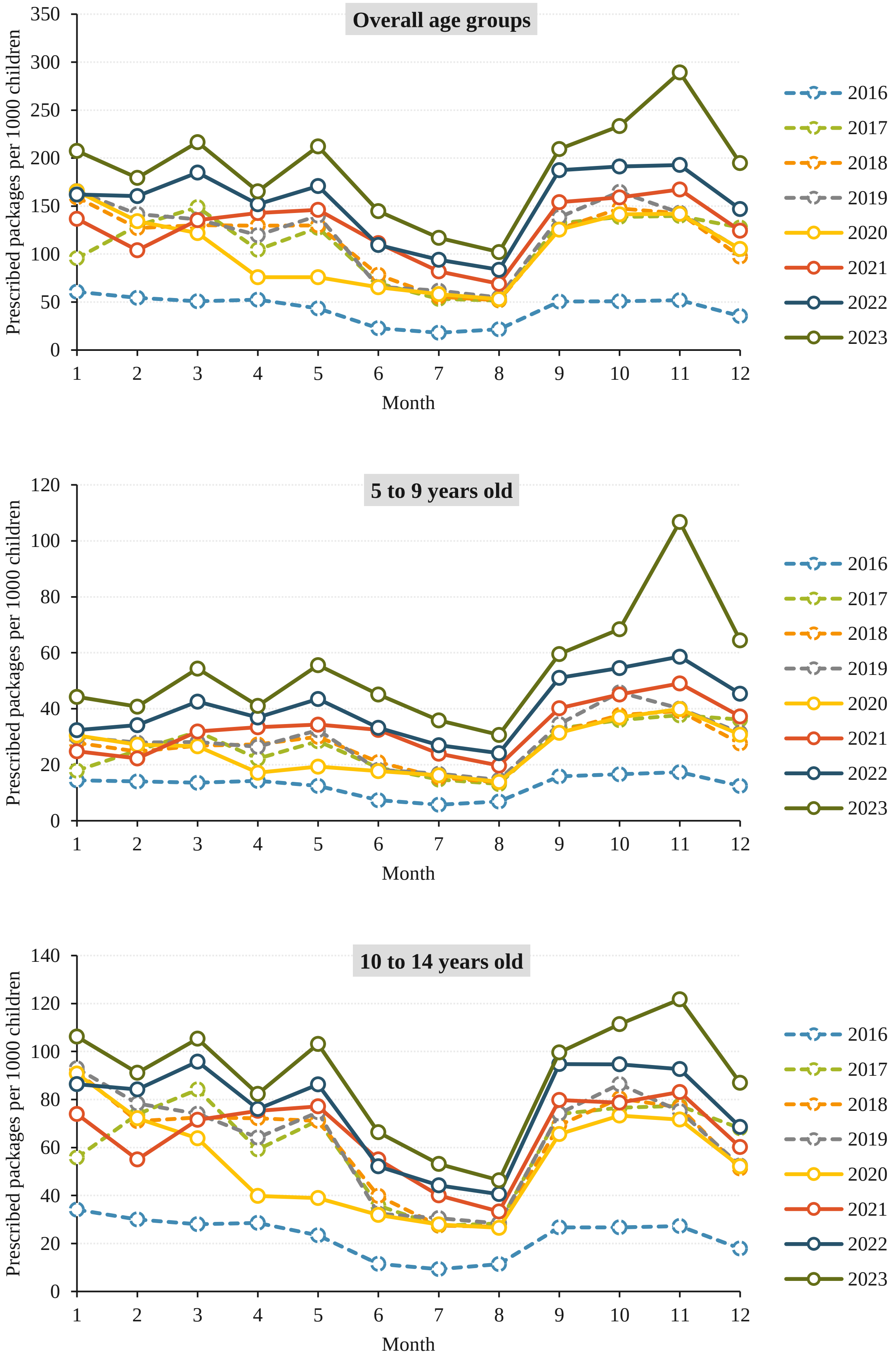

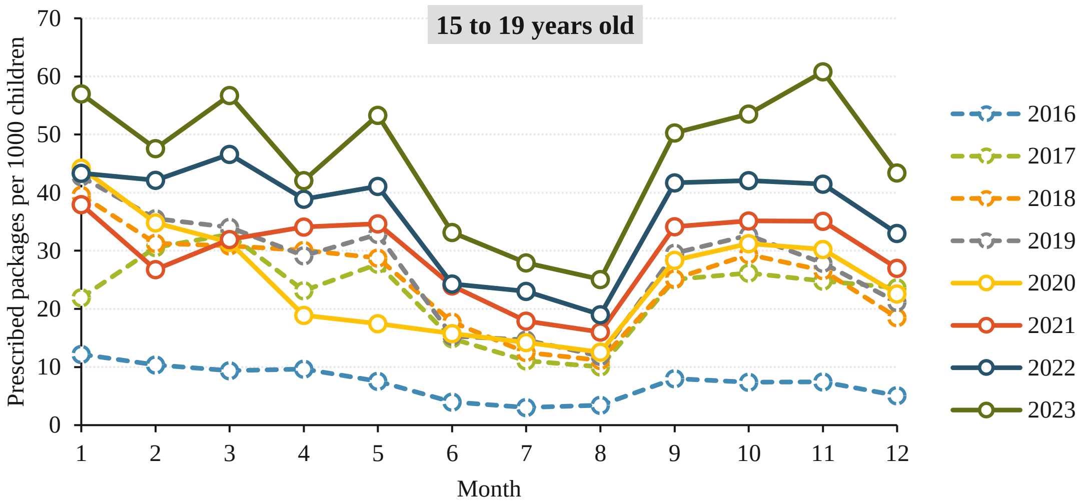

The dashed lines represent pre-COVID-19 years.

Supplementary Information Table 1. Descriptive statistics for monthly rates (prescribed packages per 1000 children/adolescents), per gender and age group, standard deviation (STD), 95% confidence intervals (CI) and the percentage change during preventive and protective policies (change point 1) and after the withdraw of the restrictive policies (change point 2).

Packages per month [x]	Mean	STD	95% CI	% Change
Girls aged 5 to 9				
Before COVID-19 Pandemic [a]	6.2	2.6	(5.4; 6.9)	
First change point [b]	6.5	2.2	(5.2; 7.7)	4.9
Second change point [c]	11.6	4.8	(9.8; 13.3)	85.7
Boys aged 5 to 9				
Before COVID-19 Pandemic [a]	17.7	7.2	(15.7; 19.8)	
First change point [b]	19.4	6.1	(15.9; 23.0)	9.7
Second change point [c]	32.6	11.8	(28.4; 36.8)	80.1
Girls aged 10 to 14				
Before COVID-19 Pandemic [a]	13.9	6.7	(12.0; 15.8)	
First change point [b]	13.9	5.1	(10.9; 16.8)	-0.4
Second change point [c]	21.1	6.8	(18.7; 23.6)	52.0
Boys aged 10 to 14				
Before COVID-19 Pandemic [a]	39.8	18.7	(34.4; 45.1)	
First change point [b]	41.0	13.4	(33.2; 48.7)	3.0
Second change point [c]	56.9	16.4	(51.0; 62.8)	42.2
Girls aged 15 to 19				
Before COVID-19 Pandemic [a]	5.9	3.1	(5.0; 6.8)	
First change point [b]	7.6	2.5	(6.1; 9.0)	28.6
Second change point [c]	12.7	4.3	(11.1; 14.2)	102.4
Boys aged 15 to 19				
Before COVID-19 Pandemic [a]	15.5	8.1	(13.2; 17.8)	
First change point [b]	17.7	5.7	(14.4; 21.0)	14.4
Second change point [c]	25.2	7.9	(22.4; 28.1)	58.0

[x] 1000 children/adolescents. [a] from January 2016 to February 2020. [b] from March 2020 to April 2021. [c] from May 2021 to December 2023 (percentage change compared with the period from January 2016 to April 2021).
